# Spontaneous Pregnancy After Surgical Correction of a Prostatic Utricle Cyst and Recurrent Varicocele: A Case Report

**DOI:** 10.1155/criu/5477079

**Published:** 2026-10-08

**Authors:** Caio Bosquê Hidalgo Ribeiro, Luiz Felipe Lessa Ortiz, Júlia Ribeiro Rezende, Bárbara Bomfim Muniz Moraes, Henrique Cunha Vieira, Amanda Lino De Faria Lessa

**Affiliations:** ^1^ GAVVA Clinic–Reproductive Medicine and Oncofertility Sao Paulo Brazil

**Keywords:** case report, ejaculatory duct obstruction, hypospermia, male infertility, prostatic utricle cyst, varicocele

## Abstract

**Background:**

Prostatic utricle cysts are rare congenital anomalies arising from incomplete regression of the Müllerian ducts that may cause partial or complete ejaculatory duct obstruction, leading to hypospermia, abnormal semen parameters and even azoospermia in severe cases. Recurrent varicocele after previous surgical repair represents an additional challenge in the management of male infertility. Here, we report a case of spontaneous pregnancy achieved after combined surgical correction of a prostatic utricle cyst and recurrent left‐sided varicocele in a couple not yet meeting the formal 12‐month criteria for infertility, illustrating the diagnostic and therapeutic considerations relevant to this clinical scenario.

**Case Presentation:**

A 27‐year‐old man with a history of bilateral microsurgical varicocelectomy in 2018, persistent hypospermia (ejaculate volume 0.1–0.5 mL), reduced sperm motility (8%–24% progressive motility) and abnormal strict Kruger morphology (1%–3%) was evaluated together with his 35‐year‐old partner after 6 months of attempting natural conception. Scrotal Doppler ultrasonography confirmed recurrent left‐sided varicocele, and pelvic magnetic resonance imaging revealed a prostatic utricle cyst measuring 1.6 × 1.0 cm. The female partner had no relevant abnormalities on complete gynaecological evaluation. The male partner underwent transurethral resection of the prostatic utricle cyst combined with subinguinal microsurgical redo varicocelectomy in a single surgical session without complications. Two months postoperatively, spontaneous clinical pregnancy was confirmed, and two semen analyses performed 4 months after surgery showed normalisation of all seminal parameters.

**Conclusions:**

This case highlights the importance of comprehensive andrological evaluation, including imaging studies, in couples with a pre‐existing, known male‐factor abnormality, even when formal infertility criteria are not yet met. Prostatic utricle cysts should be considered in the differential diagnosis of obstructive male infertility, particularly in patients with unexplained hypospermia. In this case, combined correction of coexisting reproductive tract abnormalities was followed by normalisation of postoperative semen parameters and a favourable reproductive outcome; because semen analysis was performed only after conception had already occurred, a causal relationship with the pregnancy itself cannot be definitively established, but the findings support a positive physiological effect of surgery. Surgical reintervention for recurrent varicocele remains justified in selected patients with persistently abnormal semen parameters.

## 1. Background

Infertility affects an estimated 10%–15% of couples of reproductive age, with male factors contributing to approximately 40%–50% of cases, either in isolation or in association with female factors [1–4]. Among male infertility aetiologies, obstructive causes—resulting from congenital or acquired abnormalities of the epididymis, vas deferens or ejaculatory ducts—are characterised by preserved spermatogenesis despite impaired sperm transport and require a diagnostic approach based on clinical, laboratory and imaging findings [4–6].

The prostatic utricle cyst is a congenital Müllerian duct remnant that may compress or obstruct the ejaculatory ducts. Although frequently asymptomatic, it may present with infertility, genitourinary infections, haematospermia or ejaculatory disorders, and early recognition may allow therapeutic intervention with a positive impact on reproductive prognosis [6–8].

Varicocele, the most common surgically correctable cause of male infertility, predominates on the left side and impairs spermatogenesis through increased scrotal temperature, oxidative stress and venous stasis [9–11]. Varicocelectomy may improve semen parameters in selected patients, but recurrence remains a clinical challenge, related to persistent or newly formed venous collaterals, technique‐dependent factors or anatomical complexity [12, 13].

Here, we describe a couple in which the male partner presented with persistent hypospermia and abnormal semen parameters secondary to the coexistence of a prostatic utricle cyst and recurrent left‐sided varicocele, detailing the diagnostic workup, combined surgical management and reproductive outcome and discussing aspects relevant to the management of male infertility.

## 2. Case Presentation

### 2.1. Clinical Background

A heterosexual couple—a 27‐year‐old man and a 35‐year‐old woman—presented after 6 months of attempts at natural conception. Although the couple had not yet reached the formal 12‐month definition of infertility, evaluation was pursued earlier because of a pre‐existing, previously documented male‐factor abnormality (persistent hypospermia and abnormal semen parameters since 2018, unresponsive to prior varicocelectomy) together with the female partner′s age of 35 years—recognised indications for expedited, rather than routine, early assessment [4, 14]. The aim was to optimise the couple′s chances of natural conception and improve reproductive prognosis; this approach is not intended to advocate universal early surgical intervention in couples without known risk factors.

### 2.2. Male Evaluation

The male partner had a personal history of depressive disorder managed with continuous antidepressant therapy since adolescence. His surgical history was significant for bilateral microsurgical varicocelectomy performed in 2018 due to previously identified abnormal semen parameters, without significant improvement following surgery. A long‐standing complaint of low ejaculatory volume since the onset of sexual activity was noted, with no other associated symptoms. The patient denied any history of testosterone replacement therapy or anabolic–androgenic steroid use.

Physical examination revealed bilateral subinguinal scars and marked dilation of the pampiniform plexus on the left side visible at rest, and on the right side evident only during Valsalva manoeuvres, with no other abnormalities of the epididymides or vas deferens. Testicular volumes are reported below on scrotal Doppler ultrasonography.

Complementary hormonal evaluation showed FSH, LH, total testosterone and oestradiol within normal reference ranges. Serial semen analyses performed over several years demonstrated persistent hypospermia (ejaculate volume ranging from 0.1 to 0.5 mL), preserved sperm concentration (16.9–47.1 million/mL), reduced motility (8%–24% progressive motility) and abnormal strict Kruger morphology (1%–3%). The most recent semen analysis, performed in July 2025, showed an ejaculate volume of 0.5 mL, sperm concentration of 25.4 million/mL, 10% progressive motility and 3% strict Kruger morphology.

Scrotal Doppler ultrasonography (July 2025) demonstrated testicular volumes of 14.1 cc on the right and 13.8 cc on the left, with an increased‐calibre venous plexus and persistent venous reflux on Doppler during the Valsalva manoeuvre on the left side, confirming recurrent varicocele. Pelvic magnetic resonance imaging (July 2025) revealed a prostatic utricle cyst measuring 1.6 × 1.0 cm, with the remaining prostatic morphology, seminal vesicles and vas deferens unremarkable for age.

### 2.3. Female Evaluation

The female partner had no relevant medical history, regular menstrual cycles, no prior pregnancies and no gynaecological complaints such as dysmenorrhoea, dyspareunia or abnormal uterine bleeding. Complementary evaluation showed an anti‐Müllerian hormone level of 0.9 ng/mL. Transvaginal ultrasonography performed at the first consultation revealed an antral follicle count of 14, with no abnormalities of the uterine cavity or ovaries. Hysterosalpingography demonstrated a normal uterine cavity, bilateral tubal patency and absence of contrast retention.

### 2.4. Management and Outcome

Based on the clinical and imaging findings, the male partner underwent combined surgical correction in a single anaesthetic session in September 2025, without immediate complications.

Transurethral resection of the prostatic utricle cyst was performed using a resectoscope under direct endoscopic visualisation (Figure S1). The procedure was performed endoscopically under direct cystoscopic visualisation. After identification of the verumontanum and the prostatic utricle cyst, complete excision of the cyst was achieved using a bipolar resectoscope with an electrosurgical resection loop. Careful resection was performed under continuous visualisation to preserve the surrounding anatomical structures, particularly the external urinary sphincter and the ejaculatory duct orifices. Complete unroofing and excision of the cyst wall were accomplished without the need for conversion or additional instrumentation. There were no intraoperative complications, including significant bleeding, limited working space or injury to adjacent structures. The procedure was completed uneventfully. The excised cystic tissue was sent for histopathological evaluation, which demonstrated a cyst wall lined by columnar to cuboidal epithelium, focally transitioning to urothelium near the verumontanum, supported by a thin fibromuscular stroma, without evidence of glandular atypia, dysplasia or malignancy—findings consistent with a benign prostatic utricle (Müllerian duct) cyst. Left‐sided subinguinal microsurgical redo varicocelectomy was performed in the same surgical session.

Two months after the procedure, in November 2025, the female partner achieved a spontaneous clinical pregnancy. Two postoperative semen analyses, performed 4 months after surgery, showed normal parameters in both samples, with normalisation of ejaculate volume, progressive motility and strict Kruger morphology compared with the preoperative assessment. An obstetric transvaginal ultrasound, performed approximately 3 months after the surgical procedure (last menstrual period 22 October 2025; menstrual gestational age 6 weeks and 0 days), confirmed a single ongoing intrauterine pregnancy, with a topical gestational sac of regular contours measuring 25.9 × 19.6 × 7.5 mm (mean gestational sac diameter 17.7 mm), a yolk sac of normal appearance measuring 3.5 mm in diameter, a live embryo with a crown‐rump length of 8.4 mm and a fetal heart rate of approximately 124 beats per minute, and predominantly diffuse trophoblastic insertion. The cervix was of normal appearance, with a closed internal os and a length of 40.2 mm, and both ovaries were topical, with normal shape, contours and dimensions, with a corpus luteum in the left ovary. Gestational age by current biometry was estimated at 6 weeks and 5 days, concordant with the menstrual dating of 6 weeks and 0 days. Written informed consent for publication of this case report was obtained from both partners.

## 3. Discussion and Conclusions

These findings support the value of systematic andrological investigation in couples with a pre‐existing, previously documented male‐factor abnormality, even when the couple has not yet reached the formal 12‐month definition of infertility [4, 14]. In this patient, persistent hypospermia—present since the onset of sexual activity and unresponsive to a prior varicocelectomy—raised the suspicion of genital tract obstruction, which was subsequently confirmed by the identification of a prostatic utricle cyst on pelvic MRI.

Although rare and usually asymptomatic, prostatic utricle cysts can cause partial or complete ejaculatory duct obstruction, resulting in low ejaculatory volume, abnormal semen parameters and, in severe cases, azoospermia [6, 15]. Transurethral resection has been associated with improvement in ejaculatory symptoms and semen parameters, and with increased rates of natural conception in selected patients, supporting the relevance of this condition in the differential diagnosis of obstructive male infertility [16, 17].

Similar findings have been reported in the literature. Zheng et al. [18] described azoospermia due to partial ejaculatory duct obstruction despite preserved testicular function and a normal hormonal profile—closely mirroring the presentation in this case—with reappearance of sperm in the ejaculate after endoscopic treatment. Jin Ye et al. [19] similarly identified an ejaculatory duct opening within a prostatic utricle cyst intraoperatively, confirming the capacity of these lesions to cause partial or functional seminal tract obstruction and supporting endoscopic unroofing or transurethral resection as an effective, low‐morbidity, fertility‐preserving approach.

Outcome data support this approach. Schroeder‐Printzen et al. [20], in a prospective series of 16 infertile men undergoing transurethral resection or incision of the ejaculatory ducts, reported the best results in patients with central midline (Müllerian duct/prostatic utricle) cysts, with postoperative patency and semen parameter improvement in up to 85% of cases, although spontaneous pregnancy rates remained modest (20%–30%). More recent systematic reviews confirm TURED as the most extensively studied approach for ejaculatory duct obstruction, with semen parameter improvement in 60%–83% of cases and spontaneous pregnancy rates of 12%–31%, particularly favourable in partial, cystic obstruction such as that observed in this patient [21, 22].

Persistence of left‐sided varicocele after prior surgical repair justified reintervention given the persistently abnormal semen parameters. Varicocele recurrence after surgical treatment is well documented (reported rates ranging from less than 5% to over 30%, depending on technique), most often related to unligated accessory or collateral spermatic veins in nonmicrosurgical approaches. Subinguinal microsurgical varicocelectomy, used in this case, offers the lowest recurrence rates (1%–3%) and is considered the gold standard for both primary and recurrent repair [9, 23, 24].

The coexistence of varicocele with other reproductive tract abnormalities, such as ejaculatory duct obstruction, may exert a synergistic negative effect on fertility. Combined correction of both abnormalities in this patient is a plausible contributor to the favourable reproductive outcome observed, although, as discussed below, this cannot be proven with the available data.

As with any single‐case description, this report has inherent limitations. Postoperative semen analyses were performed only after conception had already occurred (4 months after surgery); although both samples showed normalisation of ejaculate volume, progressive motility and strict morphology—providing objective evidence of a favourable physiological effect of surgery on semen parameters—these data cannot demonstrate that semen quality had already improved at the time conception occurred. Coincidental natural conception or unmeasured female‐factor variability therefore cannot be entirely excluded as contributors to the observed pregnancy. The temporal association between surgical correction and spontaneous pregnancy, although supported by the subsequent normalisation of semen parameters, does not by itself establish definitive causality and should be interpreted with appropriate caution.

Overall, this case reinforces the value of detailed andrological evaluation—including advanced imaging—in couples with a pre‐existing, previously documented male‐factor abnormality, even before the couple formally meets the 12‐month definition of infertility. Prostatic utricle cysts should be considered in the differential diagnosis of obstructive male infertility, and recurrent varicocele may exert an additional negative impact on semen parameters, potentially justifying surgical reintervention in selected patients. In this case, combined correction of both abnormalities was followed by spontaneous pregnancy and, subsequently, by normalisation of semen parameters on postoperative analysis; although this sequence does not by itself prove that the pregnancy resulted directly from the surgical intervention, it lends objective support to a favourable physiological effect of the combined correction. An individualised, couple‐centred approach remains warranted in similar clinical scenarios.

**Nomenclature**
AMH: anti‐Müllerian hormone
FSH: follicle‐stimulating hormone
LH: luteinising hormone
MRI: magnetic resonance imaging
TURED: transurethral resection of the ejaculatory ducts

## Funding

No funding was received for this manuscript.

## Disclosure

All authors read and approved the final version of the manuscript. Luiz Felipe Lessa Ortiz had full access to all the data in this study and takes full responsibility for the integrity of the data and the accuracy of the data analysis.

## Ethics Statement

This case report does not involve experimental research on human subjects. The patient received standard clinical care, and all clinical data were collected as part of routine medical practice. Written informed consent for publication of this case report and the accompanying clinical data and images was obtained from the patient prior to submission. According to local institutional guidelines, ethics committee approval was not required for the publication of a single case report.

## Consent

Written informed consent for publication of this case report and any accompanying images was obtained from both patients.

## Conflicts of Interest

The authors declare no conflicts of interest.

## Supporting information


**Supporting Information** Additional supporting information can be found online in the Supporting Information section. Supporting Information. Figure S1: Urethroscopic view using a resectoscope following transurethral resection of the prostatic utricle cyst, demonstrating complete unroofing of the cystic cavity with restoration of urethral patency and relief of the ejaculatory outflow obstruction. The postoperative appearance confirms adequate decompression of the prostatic utricle and preservation of the surrounding urethral anatomy.

## Data Availability

Data sharing is not applicable to this article as no datasets were generated or analysed during the current study.
